# Inverted colonic diverticulum mimicking a polyp: A case report on diagnostic challenges and clinical implications

**DOI:** 10.1016/j.ijscr.2025.111389

**Published:** 2025-05-01

**Authors:** Suha Sholi, Walla Dawood, Mohammed M.H. Hajhamad

**Affiliations:** a Department of Medicine, College of Medicine and Health Sciences, An-Najah National University, Nablus 44839, Palestine; b Department of General Surgery, An-Najah National University Hospital, Nablus 44839, Palestine; c Department of General Surgery, Rafidia Hospital, Nablus, Palestine

**Keywords:** Case report, Inverted colonic diverticulum, Colonoscopy complications, Colonic polyp, Perforation of colon

## Abstract

**Introduction and importance:**

Inverted colonic diverticulum (ICD) is a rare intraluminal lesion that typically presents with a polypoid appearance, which can make it challenging to differentiate from colonic polyps. ICDs occur in about 0.7 % to 1.7 % of individuals, with a mean age of 59 years, and a slightly higher prevalence in males.

**Case presentation:**

This case report presents a 40-year-old male with ICD that was misdiagnosed as polyp after a colonoscopy showed polypoid lesions in the context of diverticulosis and underwent polypectomy, colonoscopy completed. A few hours after the procedure, the patient developed symptoms consistent with complicated diverticulitis and required antibiotic treatment and CT-guided aspiration.

**Clinical discussion:**

This case highlights the difficulty in differentiating ICD from colonic polyps during colonoscopy. Although ICDs are benign, they can closely resemble polyps, and misidentifying them may result in unnecessary and potentially harmful interventions. Endoscopists should be aware of the distinctive features of ICDs and employ techniques like probing and air insufflation to avoid unnecessary procedures.

**Conclusion:**

Prompt recognition of ICDs can help prevent complications such as colonic perforation and unnecessary surgery, ultimately leading to improved patient outcomes.

## Highlights

• ICD is a rare finding, often mistaken for polyps, increasing risk of complications.
• Endoscopic features like “aurora rings” and maneuvers aid in accurate diagnosis.
• Colonic perforation from ICD can be hard to detect early, even with a CT scan.
• Case shows the importance of awareness to prevent harm and improve patient recovery.

## 1 Introduction

Colonic diverticular disease (DD) is a common finding during colonoscopy, characterized by outpouching of the colonic wall. It affects approximately 5 % of individuals at age 40 and increases in prevalence, reaching up to 65 % by age 80 [1]. In contrast, inverted colonic diverticulum (ICD) is a rare occurrence, found in only 0.7–1.7 % of cases, typically in individuals with a mean age of 62 years. ICDs show a slight male predominance and are most often located in the sigmoid colon [2,3].

An inverted diverticulum (ID) typically lacks a stalk, which makes it resemble a colonic polyp, posing a challenge for differentiation during colonoscopy [4]. If misidentified and subjected to endoscopic resection, the procedure can result in colonic perforation [[4], [5], [6]]. Diagnosis of colonic perforation secondary to ICD may be challenging as it may not be detected early even with CT scan. However, distinct endoscopic features and certain colonoscopic maneuvers can aid in differentiating ICDs from true polyps [5,7]. In this case, we describe a case of inverted colonic diverticulum which was mistaken as a polyp that was treated by Polypectomy, in addition to the challenges in early diagnosis of complications and emphasize the importance of accurate identification to avoid unnecessary complications.

## 2 Case presentation

**Fig. 1:**
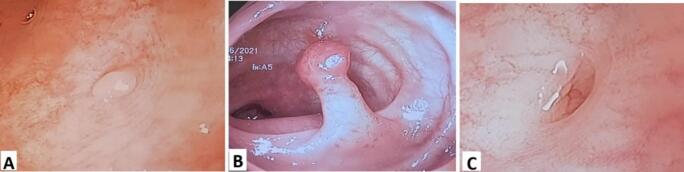
Colonoscopic view, showing a tiny polyp at the sigmoid colon (ICD) (A), pedunculated polyp less than 1 cm (B), and a diverticulum at the sigmoid colon (C).

A 40-year-old man with an unremarkable past medical history presented to the surgery clinic with intermittent lower abdominal pain and alteration in bowel motion. A computed tomography (CT) scan showed sigmoid diverticulosis along with mild wall thickening. Treated conservatively with antibiotics, and planned for elective colonoscopy; colonoscopy done after about 4 weeks, At endoscopic examination a small polyp (Fig. 1a), and a pedunculated polyp less than 1 cm (Fig. 1b), were detected at mid-sigmoid colon in the context of segmental colitis associated with diverticulosis (Fig. 1c). The ICD which was mistaken as a small polyp (Fig. 1a) was removed by piece-meal polypectomy while the pedunculated one was removed by hot-snare polypectomy. Notably, the patient was unable to tolerate the endoscope, resulting in an incomplete colonoscopy. Histopathological analysis of the removed lesions revealed the following: the pedunculated sigmoid polyp was a tubular adenoma with moderate dysplasia; the small sessile lesion (initially mistaken for polyp) showed mild chronic non-specific colitis without evidence of microorganisms, architectural distortion, dysplasia, or malignancy.

**Fig. 2:**
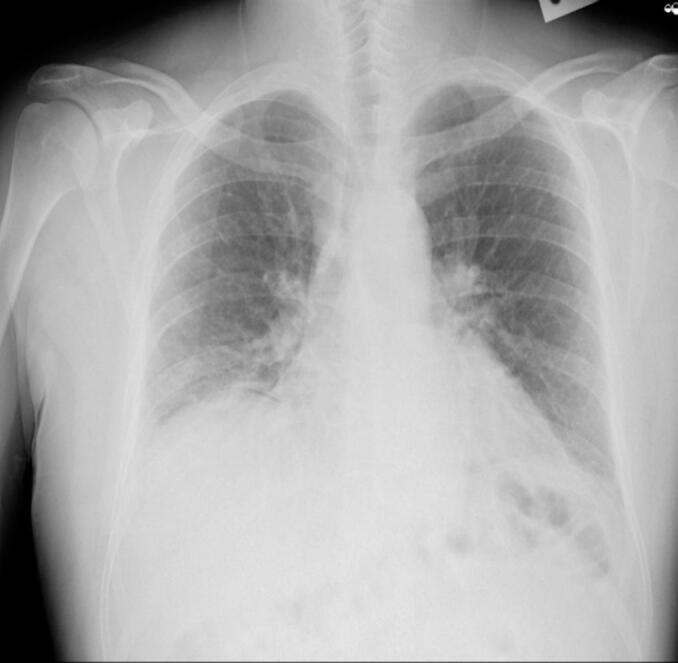
Chest X-ray showing no significant findings.

A few hours post colonoscopy the patient presented to ED with sharp left lower abdominal pain, radiating to the groin along with nausea and feverish sensation, on examination he was febrile (38.2 °C) with tenderness at the left lower quadrant. Investigations showed White Blood Cells (WBC): 10.7 k/μL (Normal range 5–11 k/μL), Neutrophils: 78 %, high C-reactive protein of 132 mg/dL (normal range 0–5 mg/dL), and the Chest x-ray (Fig. 2) and abdominal ultrasound were performed and were unremarkable.

The patient was managed with bowel rest, hydration, intravenous antibiotics and analgesia. The patient showed improvement and was discharged home.

**Fig. 3:**
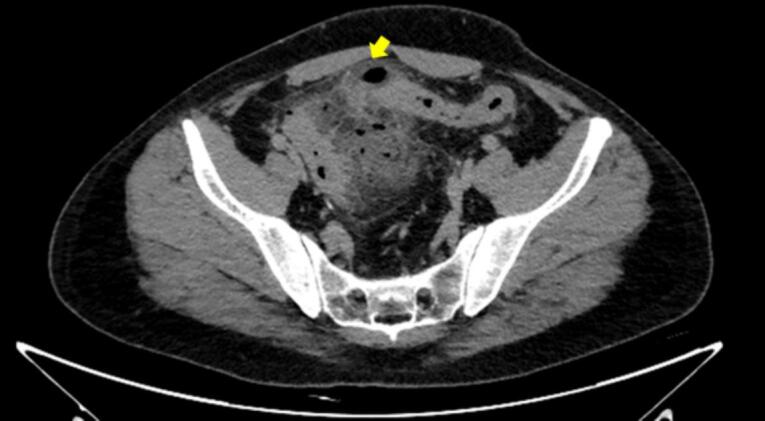
Sigmoid wall thickening with fat stranding suggestive of diverticulitis.

On the fourth-day post colonoscopy, the patient returned to ED complaining of worsening severe abdominal pain for which he was admitted and a CT scan revealed sigmoid wall thickening with fat stranding suggestive of diverticulitis. However, no pneumoperitoneum to suggest perforation (Fig. 3), and the patient was managed with intravenous antibiotics.

**Fig. 4:**
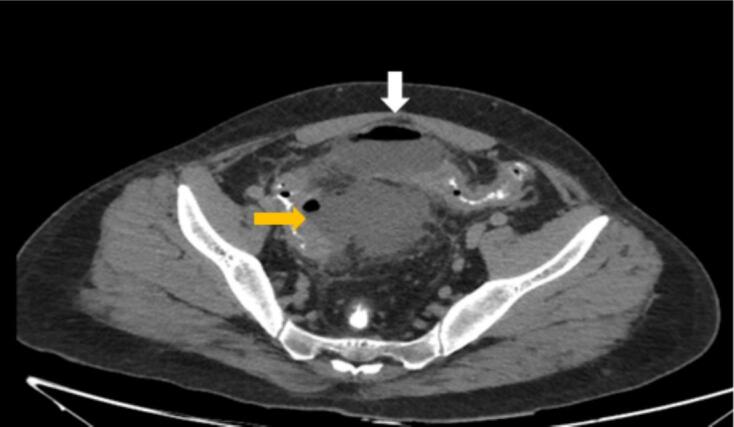
Two large collections at lower abdomen. The first collection (white arrow) 6.6 × 3.8 cm anterior to the sigmoid colon with air fluid level, originating from the colonic wall (sub serosal). Second collection (yellow arrow) measures 8 × 6.5 cm with air locules. (For interpretation of the references to colour in this figure legend, the reader is referred to the web version of this article.)

On the fifth day of admission the patient developed fever, nausea, and vomiting. The CT scan was repeated which displayed 2 large collections, the first collection (6.6 × 3.8 cm) anterior to the sigmoid colon and the second collection (8 × 6.5 cm) with air locules (Fig. 4).

These were managed with CT-guided aspiration, bowel rest, total parenteral nutrition and intravenous antibiotics.

At 6 weeks post drainage follow-up, no further abdominal pain was described and control CT scan showed resolution of the collections. And the patient was discharged home in good clinical condition.

## 3 Discussion

Inverted colonic diverticulum (ICD) is a rare intraluminal lesion that often presents with a polypoid appearance, making it difficult to distinguish from colonic polyps [[4], [5], [6]]. ICD occur in approximately 0.7 % to 1.7 % of individuals, with a mean age of 62 years, and there is a slightly higher prevalence in males [2,3]. These lesions are most commonly found in the sigmoid colon [2,3]. While they typically have a sessile morphology, pedunculated structures can also occur [5].

Patients with diverticular disease (DD) have a higher incidence of colonic polyps compared to those without diverticulosis, and as such, polypoid lesions in diverticular segments are highly likely to be ICDs [8,9]. Given their polyp-like appearance, ICDs pose a risk of being misdiagnosed during colonoscopy, leading to accidental biopsy or polypectomy, which can result in serious complications like colonic perforation [[4], [5], [6]].

ICDs begin as typical diverticula but may intermittently reverse due to changes in intraluminal and intra-abdominal pressure [5]. Distinguishing ICDs from colonic polyps during colonoscopy involves careful observation and specific colonoscopic maneuvers to confirm the diagnosis and avoid unnecessary intervention [5,7].

Endoscopic features of a suspected ICD typically include a smooth, shiny pink mucosa that is indistinguishable from the surrounding normal colonic mucosa. One key characteristic of ICDs is the presence of aurora rings, which are concentric rings surrounding the base of the lesion. These features alone can make ICDs difficult to differentiate from colonic polyps [5]. However, several endoscopic maneuvers can help distinguish ICDs from polyps. Among of which are palpation of the lesion reveals that it is soft and empty, unlike the more solid feel of a polyp, continual probing with closed biopsy forceps causes invagination of the lesion wall, creating a “radiating pillow sign.” Additionally, air insufflation can lead to partial eversion or reversal of the ICD, while a jet of water can flatten or partly everts the lesion, producing the so-called “water jet sign.” These maneuvers are critical for confirming the diagnosis of ICD and preventing unnecessary biopsy or polypectomy [5,[10], [11], [12]]. Other subtle signs and maneuvers during endoscopy to differentiate between colonic polyps versus ICDs can be identified from more recent approaches, including the use of submucosal saline injection, where ICDs typically flatten with a central dimpling due to their pseudo-diverticular nature that does not contain all layers of the bowel. This maneuver is reliable for both small and large ICD. However, a negative finding for this new maneuver cannot exclude ICD owing to the varying ICD shapes and locations. Additionally, when combined with indigo carmine dye, this maneuver may enhance the visibility of Aurora rings. These findings provide added diagnostic clues particularly useful in borderline cases where conventional signs are inconclusive [13].

## 4 Conclusion

This case highlights the challenge of distinguishing an inverted colonic diverticulum (ICD) from colonic polyps during colonoscopy. Although ICDs are benign, their resemblance to polyps can lead to misdiagnosis, resulting in unnecessary and potentially harmful interventions. Early recognition of ICDs is vital to prevent complications such as colonic perforation and avoid unwarranted surgeries, thereby improving patient outcomes. Diagnosing colonic perforation caused by ICD can be particularly difficult, as it may not be readily identified on CT scans. This underscores the importance of diagnosing complications or delayed presentations and maintaining a high index of suspicion when managing similar cases.

## Author contribution

Suha Sholi, Walla Dawood, and Mohammed Hajhamad contributed to the data collection, writing the paper. All authors have seen and approved the final version of the Abstract for publication.

This case report was prepared in accordance with the SCARE checklist for case reports [14].

## Consent

Written informed consent was obtained from the patient for publication of this case report and accompanying images. A copy of the written consent is available for review by the Editor-in-Chief of this journal on request identifying details of the patient are not essential for the case report.

## Ethical approval

Institutional Review Board (IRB) at An-Najah National University approval was waived for this case report, as per our institution's policies on single-patient case reports.

## Guarantor

Suha Sholi.

Mohammed Hajhamad.

## Research registration number

Not applicable.

## Funding

This research did not receive any specific grant from funding agencies in the public, commercial, or not-for-profit sectors.

## Conflict of interest statement

The authors declare no conflicts of interest related to this publication.
